# Supplementary material for: Superconductivity from the condensation of topological defects in a quantum spin-Hall insulator
Source: Nat Commun. 2019 Jun 14;10:2658. doi: 10.1038/s41467-019-10372-0 (PMC6572839; doi:10.1038/s41467-019-10372-0)
Supplement: Supplementary file 1 — Supplementary Information [file 41467_2019_10372_MOESM1_ESM.pdf]

Supplementary Information.
Superconductivity from the Condensation of Topological Defects in a Quantum Spin-Hall Insulator

Yuhai Liu,[1] Zhenjiu Wang,[2] Toshihiro Sato,[2] Martin Hohenadler,[2] Chong Wang,[3] Wenan Guo,[1,4] and Fakher F. Assaad[2]

[1]*Department of Physics, Beijing Normal University, Beijing 100875, China*
[2]*Institut für Theoretische Physik und Astrophysik, Universität Würzburg, Am Hubland, 97074 Würzburg, Germany*
[3]*Perimeter Institute for Theoretical Physics, Waterloo, Ontario, Canada N2L 2Y5*
[4]*Beijing Computational Science Research Center, Beijing 100193, China*

I. SUPPLEMENTARY NOTES

Observables. Symmetry-broken states are characterised by a local order parameter $\hat{\boldsymbol{O}}_{\boldsymbol{r},\boldsymbol{\delta}}$, where $\boldsymbol{r}$ denotes a unit cell and $\boldsymbol{\delta}$ an orbital within the unit cell. The associated time-displaced correlation functions read

$$S^{O}_{\boldsymbol{\delta},\boldsymbol{\delta}'}(\boldsymbol{q},\tau) = \frac{1}{L^2}\sum_{\boldsymbol{r},\boldsymbol{r}'}\langle \hat{\boldsymbol{O}}_{\boldsymbol{r},\boldsymbol{\delta}}(\tau)\cdot\hat{\boldsymbol{O}}_{\boldsymbol{r}',\boldsymbol{\delta}'}(0)\rangle e^{i\boldsymbol{q}\cdot(\boldsymbol{r}-\boldsymbol{r}')} \,. \tag{1}$$

For the finite-size scaling analysis, we consider the order parameter

$$m^O = \sqrt{\frac{\Lambda_1(S^{O}_{\boldsymbol{\delta},\boldsymbol{\delta}'}(\boldsymbol{0},0))}{L^2}}\,, \tag{2}$$

the equal-time correlation ratios

$$R^O = 1 - \frac{\Lambda_1(S^{O}_{\boldsymbol{\delta},\boldsymbol{\delta}'}(\Delta\boldsymbol{q},0))}{\Lambda_1(S^{O}_{\boldsymbol{\delta},\boldsymbol{\delta}'}(\boldsymbol{0},0))}\,, \tag{3}$$

with $|\Delta\boldsymbol{q}| = \frac{4\pi}{\sqrt{3}L}$, the susceptibilities

$$\chi^O = \Lambda_1\left(\int_0^\beta d\tau S^{O}_{\boldsymbol{\delta},\boldsymbol{\delta}'}(\boldsymbol{0},\tau)\right)\,, \tag{4}$$

and the corresponding correlation ratios

$$R^O_\chi = 1 - \frac{\Lambda_1\left(\int_0^\beta d\tau S_{\boldsymbol{\delta},\boldsymbol{\delta}'}(\Delta\boldsymbol{q},\tau)\right)}{\Lambda_1\left(\int_0^\beta d\tau S^{O}_{\boldsymbol{\delta},\boldsymbol{\delta}}(\boldsymbol{0},\tau)\right)}\,. \tag{5}$$

Here, $\Lambda_1()$ indicates the largest eigenvalue of the corresponding matrix in orbital space (6×6 for spin currents, 2×2 for pairing) and the ordering wave vector is at the Γ point.

These quantities exhibit the following finite-size scaling behaviour near the critical point:

$$\begin{aligned} m^O(L,\lambda) &= L^{(2-d-z-\eta)/2} f_1(L^z/\beta,(\lambda-\lambda_c)L^{1/\nu},L^{-\omega_1})\,,\\ R^O(L,\lambda) &= f_2(L^z/\beta,(\lambda-\lambda_c)L^{1/\nu},L^{-\omega_2})\,,\\ \chi^O(L,\lambda) &= L^{2-\eta} f_3(L^z/\beta,(\lambda-\lambda_c)L^{1/\nu},L^{-\omega_3})\,,\\ R^O_\chi(L,\lambda) &= f_4(L^z/\beta,(\lambda-\lambda_c)L^{1/\nu},L^{-\omega_4})\,. \end{aligned} \tag{6}$$

Here, λ_c, ν, η, and z are the critical coupling, the correlation length exponent, the anomalous dimension, and the dynamical critical exponent, respectively.

The correlation ratios R^O and R^O_χ are both renormalization group (RG) invariant quantities at the critical point and hence provide a simple way to estimate λ_c and ν without any knowledge about η. However, the generic corrections-to-scaling exponent ω is not necessarily the same for all four quantities in equation (6). Such corrections generally arise from irrelevant operators of the fixed point and the analytic part of the free energy. If the absolute value of the negative RG dimension is relatively large, the main contribution to ω will come from the background term of the free energy [1]. In this case,

$$\omega_1 = \omega_2 = 2 - z - \eta, \quad \omega_3 = \omega_4 = 2 - \eta\,. \tag{7}$$

This suggests that the susceptibility χ and the corresponding correlation ratio R_χ will have smaller scaling corrections than the corresponding equal-time quantities if the effect of the negative RG dimension is small at λ_c.

We assumed a dynamical critical exponent $z = 1$ for both the SM-QSH and the QSH-SC transition. This is motivated by the Lorentz invariance of the corresponding field theories [2, 3]. Accordingly, in our simulations, we used $\beta = L$ and thereby fixed L^z/β in the above finite-size scaling expressions.

Trotter decomposition used for the QMC simulations. For the finite-temperature QMC simulations underlying this work, imaginary time was discretized with a spacing $\Delta\tau = \beta/L_\tau$. To ensure hermiticity, the partition function is written as

$$Z = \mathrm{Tr}\left[e^{-\frac{\Delta\tau}{2}\hat{H}_t} \left(\prod_{i=1}^{N} e^{-\frac{\Delta\tau}{2}\hat{H}^x_\lambda(i)} e^{-\frac{\Delta\tau}{2}\hat{H}^y_\lambda(i)} e^{-\frac{\Delta\tau}{2}\hat{H}^z_\lambda(i)} \prod_{j=N}^{1} e^{-\frac{\Delta\tau}{2}\hat{H}^z_\lambda(j)} e^{-\frac{\Delta\tau}{2}\hat{H}^y_\lambda(j)} e^{-\frac{\Delta\tau}{2}\hat{H}^x_\lambda(j)} \right) e^{-\frac{\Delta\tau}{2}\hat{H}_t} \right]^{L_\tau} \tag{8}$$

where $\hat{H}_t$ is defined by equation (1) and the interaction (2) was partitioned into local operators $H^\alpha_\lambda(i)$ acting on spin component $\alpha = x, y, z$ and on hexagon i. The leading discretization error for the partition function then scales as $\Delta\tau^2$.

The Trotter decomposition in equation (8) breaks the global SU(2) spin rotation symmetry. For example, $[\hat{H}^x_\lambda(i), \hat{H}^y_\lambda(i)] \neq 0$, so that equation (8) will not be invariant under a global SU(2) rotation. Because SU(2) symmetry breaking is a relevant perturbation for both critical points considered, care has to be taken to ensure that its effects, which scale as $\Delta\tau^2$, remain below the relevant energy scale. An explicit test involves the total spin operator and generator of global SU(2) rotations

$$\boldsymbol{S}_{\text{tot}} = \frac{1}{L}\sum_{\boldsymbol{r},\boldsymbol{\delta}} \boldsymbol{S}_{\boldsymbol{r},\boldsymbol{\delta}} \,. \tag{9}$$

Here, $\boldsymbol{S}_{\boldsymbol{r},\boldsymbol{\delta}} = \hat{\boldsymbol{c}}^\dagger_{\boldsymbol{r}+\boldsymbol{\delta}} \boldsymbol{\sigma} \boldsymbol{c}_{\boldsymbol{r}+\boldsymbol{\delta}}$ and $\boldsymbol{\delta}$ runs over the positions of atoms in the unit cell at $\boldsymbol{r}$. Supplementary Fig 1 shows the associated time-displaced spin-spin correlation function. A global SU(2) spin symmetry implies that this quantity is independent of imaginary time. The numerical results are essentially constant in imaginary time if the symmetric Trotter decomposition is used. Therefore, the latter was employed together with $\Delta\tau = 0.2$ for all results of this work.

II. SUPPLEMENTARY METHODS

Finite-size scaling analysis by different crossing points. In addition to the main text, where a 'two-size crossing' with sizes L and $L+6$ was performed, we provide a cross check based on crossings of L and $L+3$.

When considering the L and $L+6$ crossings in the main text for the estimation of λ^{SC}_{c2} (see Fig. 3 of the main text), we are obliged to take into account the $L = 6$ data. Upon inspection, the fit turns out to be rather bad since $\chi^2/\text{DOF} = 66.9$. On the other hand, if we consider the L and $L+3$ crossing points, we can omit the $L = 6$ data and get a more acceptable $\chi^2/\text{DOF} = 6.8$. As apparent from Supplementary Fig. 3, the extrapolated value of λ^{SC}_{c2} based on the crossing points of L and $L+3$ compares favourably with the analysis in the main text.

Supplementary Fig 2**a**,**b** show the crossing values of λ and $1/\nu$ at the semimetal-QSH transition, as obtained from the correlation ratio. Supplementary Fig 2 **c** shows the anomalous dimension η at each crossing point, as well as a fit based on an expression analogous to Eq. (6) of the main text,

$$\eta^O(L) = 2 - \frac{1}{\log r} \log\left(\frac{\chi^O(\lambda, rL)}{\chi^O(\lambda, L)} \right)\Bigg|_{\lambda=\lambda^O_c(L)} , \tag{10}$$

where $r = \frac{L+3}{L}$.

The results of a similar analysis for the QSH-SC transition are reported in Supplementary Fig. 3.

Consistency of the finite-size scaling analysis. As an independent consistency check on the results from the 'two-size crossing' method used in the main text, we consider in this section a collective fitting of multiple system sizes.

The collective fitting of λ_c and ν is based on a polynomial expansion of the scaling function of $R^O_\chi(L, \lambda)$ in equation (6). Taking $\beta = L$, we have

$$R^O_\chi(L, \lambda) \approx \sum_{p=0}^{n} a_p (\lambda - \lambda_c)^p L^{p/\nu} + L^{-\omega} \sum_{q=0}^{m} b_q (\lambda - \lambda_c)^q L^{q/\nu} \,. \tag{11}$$

DQCP – QSH					
L_{min}	λ_c	$R_\chi^{\text{QSH}}(\lambda_c)$	$1/\nu$	ω	χ^2/DOF
9	0.03332(1)	0.8665(4)	2.22(5)	n/a	139/44
12	0.03326(2)	0.8702(8)	2.21(7)	n/a	58.9/32
15	0.03326(3)	0.870(2)	2.3(2)	n/a	33.4/22
18	0.03321(5)	0.875(5)	2.4(3)	n/a	13.9/12
9	0.3314(5)	0.89(2)	1.55(9)	0.9(3)	29.1/41
12	0.331(2)	0.92(9)	1.5(2)	0.4(9)	24.7/29
15	0.331(2)	0.89(2)	1.6(3)	4(3)	15.1/19
9	0.3315(5)	0.89(2)	1.5(2)	0.9(4)	28.2/36
12	0.331(2)	0.91(8)	1.5(2)	0.5(9)	23.8/26
15	0.331(2)	0.89(3)	1.7(3)	3(3)	13.9/16

DQCP – SC					
L_{min}	λ_c	$R_\chi^{\text{SC}}(\lambda_c)$	$1/\nu$	ω	χ^2/DOF
9	0.032675(4)	0.8592(4)	1.32(3)	n/a	1239/40
12	0.032791(5)	0.8742(5)	1.60(4)	n/a	123/29
15	0.032843(8)	0.882(1)	1.83(6)	n/a	15.9/18
18	0.03286(3)	0.884(4)	1.9(2)	n/a	2.23/9
9	0.03296(5)	0.907(9)	2.1(2)	1.7(3)	53.8/37
12	0.03287(2)	0.887(2)	2.3(2)	4.4(8)	26.0/26
15	0.0329(2)	0.89(3)	2.0(4)	3(13)	15.1/15
9	0.0331(2)	0.94(4)	1.9(2)	1.1(5)	42.5/33
12	0.03285(3)	0.884(5)	2.0(3)	8(6)	20.6/22
15	0.0329(2)	0.89(3)	1.7(5)	4(16)	14.4/13

Supplementary Table 1. Collective fitting at the DQCP for λ_c, $1/\nu$, and $R_\chi^O(\lambda_c)$ based on the correlation ratios $R_\chi^O(L,\lambda)$ and equation (11). We compare the case without taking into account scaling corrections (data rows 1–4) to the case with $m = 1$ (rows 5–7 correspond to a larger data window, rows 8–10 to a smaller one); in both cases, $n = 2$. Reported errors correspond to standard errors.

DQCP – QSH		
L_{min}	η	χ^2/DOF
6	0.30(2)	990/68
9	0.24(1)	249/57
12	0.19(2)	92.0/40
15	0.24(3)	49.8/25
18	0.18(6)	12.0/12

DQCP – SC		
L_{min}	η	χ^2/DOF
6	0.344(8)	525/45
9	0.308(7)	128/36
12	0.28(2)	65.6/26
15	0.23(2)	24.2/17
18	0.19(9)	7.47/8

Supplementary Table 2. Same analysis as in table 1 but for the exponent η using equation (12). Here $n = 2$, while scaling corrections are ignored. Reported errors correspond to standard errors.

Here, n and m are the expansion orders for the dimensionless and the scaling correction part of the universal function, respectively.

Table 1 reports the results of fits for the two order parameters at the DQCP, including the case with $m = 1$, as well as the case without considering any scaling correction. We set $n = 2$ for all the fits. For $m = 1$, the fitting of the QSH correlation ratio is satisfactory in terms of χ^2/DOF for $L_{\text{min}} \geq 9$, and the results are consistent with each other for $L_{\text{min}} = 9, 12, 15$. Taking $\lambda_{c2} = 0.03314(5)$ and $1/\nu = 1.55(9)$ from the fit with $L_{\text{min}} = 9$, we get consistency with $\lambda_{c2}^{\text{QSH}} = 0.03322(3)$ and $1/\nu^{\text{QSH}} = 1.7(4)$ from the 'two-size crossing' analysis in the main text. The results of a fit with a smaller data window are shown in the last three rows of table 1, revealing that the results are stable upon variation of the number of degrees of freedom. A fit using R_χ^{SC} also produces acceptable values of χ^2/DOF for $L_{\text{min}} = 9$, 12 and 15 and compares favourably with the results presented in the main text.

To reduce the number of degrees of freedom in the fit of the anomalous dimension η, a substitution in terms of the scaling form for χ^O and R_χ^O in equation (6) is performed, using the expansion (we ignore the correction-to-scaling term)

$$\chi^O(L,R) = L^{2-\eta} f(R) \approx L^{2-\eta} \sum_{p=0}^{n} a_p R^p \,. \tag{12}$$

As shown in table 2, the collective fitting for both order parameters is acceptable for $L_{\text{min}} \geq 12$. The corresponding values $\eta^{\text{QSH}} = 0.194(9)$ and $\eta^{\text{SC}} = 0.279(9)$ agree well with the values $0.21(5)$ and $0.22(6)$ obtained with the 'two-size crossing' approach used in the main text.

We also carried out the collective fitting at the Gross-Neveu critical point. In contrast to the DQCP, this phase transition suffers much less from corrections to scaling (as shown in Fig. 3 of the main text, the crossing points converge quickly). Hence, a fit without the scaling correction term is performed, and the results are shown in table 3. As can be seen, a good χ^2/DOF is obtained once the $L = 6$ data set is neglected, and fits for $L_{min} = 9, 12, 15$ or 18 produce consistent results. Taking $\lambda_{c1} = 0.01891(2)$ and $1/\nu = 1.17(3)$ from $L_{\text{min}} = 9$, the results match those of the analysis in the main text.

Gross-Neveu – QSH					
L_{min}	λ_c	$R_\chi^{\text{QSH}}(\lambda_c)$	$1/\nu$	ω	χ^2/DOF
6	0.01898(2)	0.6970(6)	1.26(2)	n/a	94.6/34
9	0.01891(2)	0.693(1)	1.17(3)	n/a	34.8/27
12	0.01882(4)	0.687(2)	1.17(5)	n/a	18.6/20
15	0.01870(7)	0.678(5)	1.14(11)	n/a	9.22/13
18	0.0186(2)	0.67(2)	1.3(4)	n/a	1.90/6

Gross-Neveu – QSH		
L_{min}	η	χ^2/DOF
6	0.666(7)	210/33
9	0.70(1)	111/26
12	0.76(2)	28.6/18
15	0.78(2)	4.4/12
18	0.81(6)	1.9/6

Supplementary Table 3. Same as table 1, but for the Gross-Neveu transition. Here $n = 2$, while scaling corrections are ignored. Reported errors correspond to standard errors.

Results for the anomalous dimension at the Gross-Neveu critical point are listed in table 3. The fits yield acceptable χ^2 values for $L_{\text{min}} \geq 12$. The exponent $\eta = 0.76(1)$ from $L_{\text{min}} = 12$ also matches the analysis in the main text.

III. SUPPLEMENTARY DATA

Single-particle gap and free-energy derivative across the QSH-SC transition. The single-particle gap Δ_{sp} is obtained from the single-particle Green function

$$G(\boldsymbol{k},\tau) = \frac{1}{L^2} \sum_{\boldsymbol{r},\boldsymbol{r}',\boldsymbol{\delta},\sigma} \langle \hat{c}^\dagger_{\boldsymbol{r}+\boldsymbol{\delta},\sigma}(\tau) \hat{c}_{\boldsymbol{r}+\boldsymbol{\delta},\sigma}(0) \rangle e^{\mathrm{i}\boldsymbol{k}\cdot(\boldsymbol{r}-\boldsymbol{r}')} \tag{13}$$

where $\boldsymbol{r} + \boldsymbol{\delta}$ runs over the two orbitals of the unit cell located at $\boldsymbol{r}$. The single-particle gap is minimal at the Dirac point $\mathrm{K} = (\frac{4\pi}{3}, 0)$ and is extracted by noting that asymptotically

$$G(\mathrm{K},\tau) \propto e^{-\Delta_{\text{sp}}\tau} \,. \tag{14}$$

Here, we used $\beta = 36$. Supplementary Fig 4a demonstrates that Δ_{sp} remains nonzero across the QSH-SC transition at $\lambda_{c2} \approx 0.033$.

In order to clarify nature of the QSH-SC transition, we also calculated the first partial derivative of the free energy density with respect to the coupling λ (we use the same notation as in the main text)

$$\frac{\partial F}{\partial \lambda} = -\frac{1}{L^2} \sum_{\varhexagon} \left\langle \left(\sum_{\langle\langle \boldsymbol{i},\boldsymbol{j} \rangle\rangle \in \varhexagon} \mathrm{i}\nu_{\boldsymbol{ij}} \hat{\boldsymbol{c}}^\dagger_{\boldsymbol{i}} \boldsymbol{\sigma} \hat{\boldsymbol{c}}_{\boldsymbol{j}} + \text{H.c.} \right)^2 \right\rangle . \tag{15}$$

Supplementary Fig 4b shows $\partial F/\partial\lambda$ for $\beta = L$ in the vicinity of $\lambda_{c2} \approx 0.033$. As expected for a continuous transition, we observe no sign of a jump.

IV. SUPPLEMENTARY DISCUSSION

Charged skyrmion defects of the QSH state. In Ref. [4], it was shown that when a QSH state is generated by spontaneous symmetry breaking, skyrmion defects of the vector order parameter will carry an electric charge of $Q_{\text{e}} = 2\text{e}$, leading to the relation

$$Q_{\text{e}} = 2\text{e}Q \tag{16}$$

where Q is the Pontryagin index that counts the winding of the unit vector order parameter on the sphere.

Here, we substantiate this fact in terms of an explicit calculation for a lattice model. Our starting point is the Hamiltonian

$$\hat{H} = -t \sum_{\langle \boldsymbol{ij} \rangle} \left(\hat{\boldsymbol{c}}^\dagger_{\boldsymbol{i}} \hat{\boldsymbol{c}}_{\boldsymbol{j}} + \text{H.c.} \right) + \lambda \sum_{\varhexagon} \boldsymbol{N}(\boldsymbol{x}) \cdot \left(\sum_{\langle\langle \boldsymbol{i},\boldsymbol{j} \rangle\rangle \in \varhexagon} \underbrace{\mathrm{i}\nu_{\boldsymbol{ij}} \hat{\boldsymbol{c}}^\dagger_{\boldsymbol{i}} \boldsymbol{\sigma} \hat{\boldsymbol{c}}_{\boldsymbol{j}} + \text{H.c.}}_{\equiv \hat{\boldsymbol{J}}_{\boldsymbol{ij}}} \right) , \tag{17}$$

where $\boldsymbol{N}(\boldsymbol{x}) = (N^x(\boldsymbol{x}), N^y(\boldsymbol{x}), N^z(\boldsymbol{x}))$ is a unit vector at position $\boldsymbol{x}$ corresponding to the centre of a hexagon. Since $\hat{H}$ is invariant under time reversal symmetry, $\hat{T}^{-1}\alpha\binom{\hat{c}_{i,\uparrow}}{\hat{c}_{i,\downarrow}}\hat{T} = \overline{\alpha}\binom{\hat{c}_{i,\downarrow}}{-\hat{c}_{i,\uparrow}}$, Kramers' theorem holds and stipulates that all eigenstates are doubly degenerate.

On the honeycomb lattice, the Pontryagin index is defined as

$$Q = \frac{1}{8\pi}\sum_{\boldsymbol{x}} \boldsymbol{N}(\boldsymbol{x}) \cdot (\boldsymbol{N}(\boldsymbol{x}+\boldsymbol{a}_1) - \boldsymbol{N}(\boldsymbol{x})) \times [(\boldsymbol{N}(\boldsymbol{x}) - \boldsymbol{N}(\boldsymbol{x}+\boldsymbol{a}_2)) + (\boldsymbol{N}(\boldsymbol{x}) - \boldsymbol{N}(\boldsymbol{x}-\boldsymbol{a}_1+\boldsymbol{a}_2))] \tag{18}$$

with unit vectors $\boldsymbol{a}_1 = (1, 0)$ and $\boldsymbol{a}_2 = (\frac{1}{2}, \frac{\sqrt{3}}{2})$.

For an arbitrary vector field $\boldsymbol{N}(\boldsymbol{x})$, Hamiltonian (17) does not preserve particle-hole (P-H) symmetry. For example, defining the P-H transformation as

$$\hat{P}_z^{-1}\alpha\begin{pmatrix}\hat{c}^\dagger_{\boldsymbol{i},\uparrow}\\ \hat{c}^\dagger_{\boldsymbol{i},\downarrow}\end{pmatrix}\hat{P}_z = \eta_i\overline{\alpha}\begin{pmatrix}\hat{c}_{\boldsymbol{i},\uparrow}\\ -\hat{c}_{\boldsymbol{i},\downarrow}\end{pmatrix}, \tag{19}$$

where $\eta_i = 1\,(-1)$ for $i \in$ A (B), we have

$$\begin{aligned}\hat{P}_z^{-1}\hat{J}^x_{\boldsymbol{i},\boldsymbol{j}}\hat{P}_z &= \hat{J}^x_{\boldsymbol{i},\boldsymbol{j}}\,,\\ \hat{P}_z^{-1}\hat{J}^y_{\boldsymbol{i},\boldsymbol{j}}\hat{P}_z &= \hat{J}^y_{\boldsymbol{i},\boldsymbol{j}}\,,\\ \hat{P}_z^{-1}\hat{J}^z_{\boldsymbol{i},\boldsymbol{j}}\hat{P}_z &= -\hat{J}^z_{\boldsymbol{i},\boldsymbol{j}}\,.\end{aligned} \tag{20}$$

A general P-H transformation can be written as

$$\hat{P}(\theta,\phi) = \hat{U}^{-1}(\theta,\phi)\hat{P}_z\hat{U}(\theta,\phi) \tag{21}$$

where

$$\hat{U}^{-1}(\theta,\phi)\begin{pmatrix}\hat{c}_{i,\uparrow}\\ \hat{c}_{i,\downarrow}\end{pmatrix}\hat{U}(\theta,\phi) = \begin{pmatrix}\cos(\theta/2) & -\sin(\theta/2)e^{-i\phi}\\ \sin(\theta/2)e^{i\phi} & \cos(\theta/2)\end{pmatrix}\begin{pmatrix}\hat{c}_{\boldsymbol{i},\uparrow}\\ \hat{c}_{\boldsymbol{i},\downarrow}\end{pmatrix}. \tag{22}$$

For Hamiltonian (17), it yields

$$\hat{P}^{-1}(\theta,\phi)\hat{H}(\boldsymbol{N})\hat{P}(\theta,\phi) = H(\boldsymbol{N}') \tag{23}$$

where

$$\boldsymbol{N}'(\boldsymbol{x}) = R^{-1}(\theta,\phi)\begin{pmatrix}1 & 0 & 0\\ 0 & 1 & 0\\ 0 & 0 & -1\end{pmatrix}R(\theta,\phi)\boldsymbol{N}(\boldsymbol{x}) \tag{24}$$

and

$$R(\theta,\phi) = \begin{pmatrix}\cos^2(\theta/2) - \sin^2(\theta/2)\cos(2\phi) & -\sin^2(\theta/2)\sin(2\phi) & -\sin(\theta)\cos(\phi)\\ -\sin^2(\theta/2)\sin(2\phi) & \cos^2(\theta/2) + \sin^2(\theta/2)\cos(2\phi) & -\sin(\theta)\sin(\phi)\\ \sin(\theta)\cos(\phi) & \sin(\theta)\sin(\phi) & \cos(\theta)\end{pmatrix}. \tag{25}$$

Thus, there is no generic P-H transformation that leaves this Hamiltonian invariant, unless $\boldsymbol{N}(\boldsymbol{x})$ is varied in an R^2 space (θ and ϕ can be defined such that $\sin\theta\cos\phi N_x + \sin\theta\sin\phi N_y + \cos\theta N_z = 0$). Since the transformation has a determinant of -1, the generic P-H transformation gives

$$Q(\boldsymbol{N}'(\boldsymbol{x})) = -Q(\boldsymbol{N}(\boldsymbol{x})). \tag{26}$$

The 'electric charge' refers to the number of occupied states at zero temperature, relative to half filling. The sign change of the Pontryagin index under a P-H transformation provides a natural way of understanding equation (16). Note that in contrast to a skyrmion, a 2D topological defect (such as vortex) has a vanishing Pontryagin index and carries no charge.

The argument of charged skyrmions fails when other Dirac mass terms are considered. For example, a system with fluctuations of a three component vector field Yukawa-coupled to the three antiferromagnetic mass terms does not break P-H symmetry. In this case, a skyrmion configuration with nonzero Pontryagin index does not carry electric charge.

We diagonalised Hamiltonian (17) on a honeycomb lattice with $L = 36$, setting $t = 1$ and $\lambda = 0.5$. Supplementary Fig 5 compares the density of states for a uniform field $\boldsymbol{N}(\boldsymbol{x})$ and for a 'hedgehog' configuration corresponding to a single skyrmion. On the lattice, the Pontryagin index is not quantised and we obtain $Q \approx -0.989$. The system remains gapped when one skyrmion is inserted, see Supplementary Fig. 5b. The breaking of P-H symmetry is also apparent from $D(\omega) \neq D(-\omega)$. Simple number counting shows that

$$\int_{-\infty}^{0} D(\omega) d\omega = N/2 + 2 \,, \quad \int_{0}^{\infty} D(\omega) d\omega = N/2 - 2. \tag{27}$$

Compared to the case of uniform polarisation in Supplementary Fig. 5a, an additional charge 2e is generated.

On a system with open boundary conditions, the Pontryagin index is not necessarily quantised and we can investigate how charge is transferred during the *insertion* of a skyrmion by varying the Pontryagin index from zero to one. Supplementary Fig 6 shows that the total charge is 'pumped' from 0 to 2e and we observe a step function at a non-integer value of Q. This is a consequence of the aforementioned Kramers theorem. As shown in Supplementary Fig. 7, the bulk remains gapped during this process, while the edge stays gapless. Thus, the charge 2e is pumped through the edge under insertion of a skyrmion.

V. SUPPLEMENTARY FIGURES

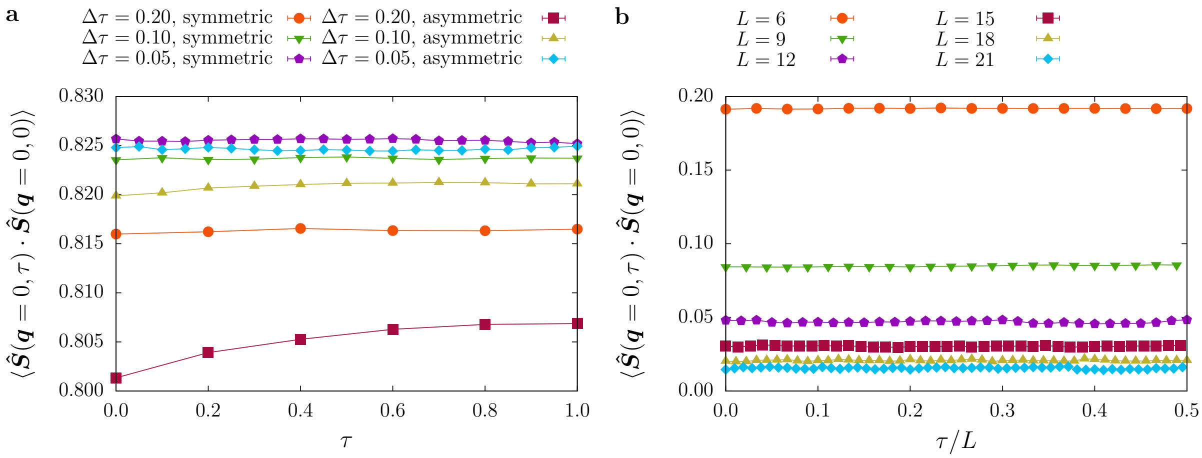

Supplementary Figure 1. **a** Time-displaced spin correlation function at $\lambda = 0.04$, $\beta = 2$, and $L = 3$. The label 'symmetric' refers to the Trotter decomposition of equation (8), whereas 'asymmetric' refers to the alternative decomposition $Z = \mathrm{Tr}\left[e^{-\Delta\tau \hat{H}_t}\left(\prod_{i=1}^{N} e^{-\Delta\tau \hat{H}^x_\lambda(i)} e^{-\Delta\tau \hat{H}^y_\lambda(i)} e^{-\Delta\tau \hat{H}^z_\lambda(i)}\right)\right]$. **b** Time-displaced spin correlation function for the symmetric Trotter decomposition and $\lambda = 0.019$, $\beta = L$. Reported error bars correspond to standard errors and are smaller than the symbol size.

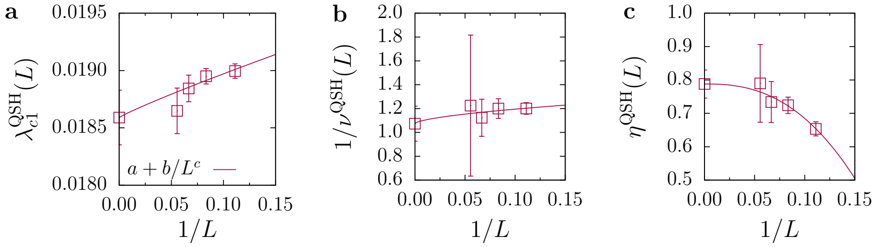

Supplementary Figure 2. **Gross-Neveu semimetal-QSH transition.** **a** Extrapolation of the crossing points of R_χ^{QSH} for L and $L+3$ gives the critical value $\lambda_{c1}^{\mathrm{QSH}} = 0.0186(3)$. **b** The inverse correlation length exponent $1/\nu^{\mathrm{QSH}} = 1.1(2)$. **c** Estimation of the anomalous dimension $\eta^{\mathrm{QSH}} = 0.79(5)$. Reported errors and error bars correspond to standard errors.

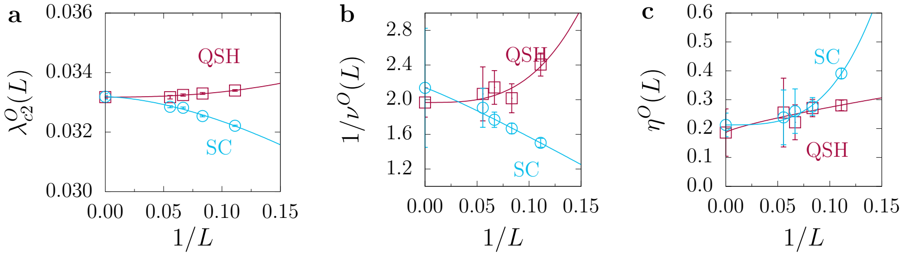

Supplementary Figure 3. **Deconfined QSH-SC transition.** **a** Estimation of the critical values $\lambda_{c2}^{\mathrm{QSH}} = 0.03317(4)$ and $\lambda_{c2}^{\mathrm{SC}} = 0.0332(1)$. **b**,**c** Critical exponents $1/\nu^{\mathrm{SC}} = 2.1(7)$, $1/\nu^{\mathrm{QSH}} = 2.0(2)$, $\eta^{\mathrm{SC}} = 0.21(5)$, and $\eta^{\mathrm{QSH}} = 0.19(9)$ from finite-size scaling of the crossing points of L and $L+3$. Reported errors and error bars correspond to standard errors.

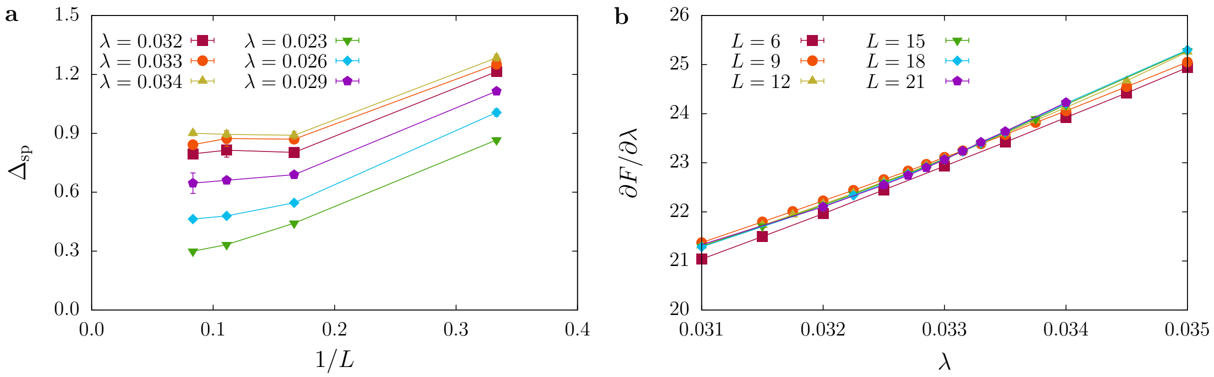

Supplementary Figure 4. **a** Fermionic single-particle gap and **b** free-energy derivative $\partial F/\partial\lambda$ across the QSH-SC transition at $\lambda_{c2}^{\mathrm{SC}} = 0.0331(3)$. Reported errors and error bars correspond to standard errors.

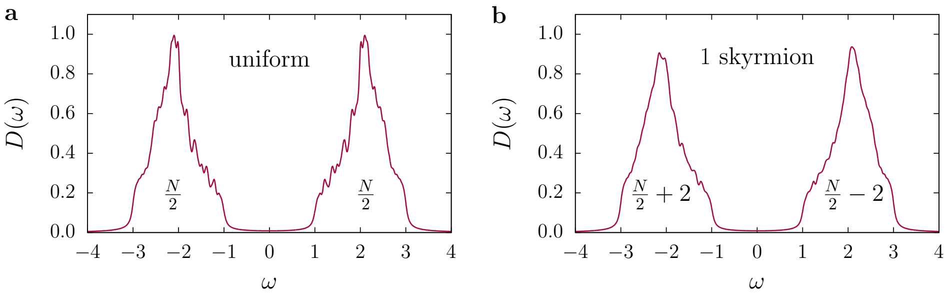

Supplementary Figure 5. Density of states $N(\omega)$ of Hamiltonian (17) for **a** uniform polarisation, **b** a 'single skyrmion' configuration with $Q \approx -0.989$. We have included an artificial broadening by using the form $D(\omega) = -\pi^{-1}\sum_n \mathrm{Im}\,(\omega - \varepsilon_n + \mathrm{i}\delta)^{-1}$, where ε_n are the eigenvalues and $\delta = 0.05$. Here, $L = 36$, $\lambda = 0.5$.

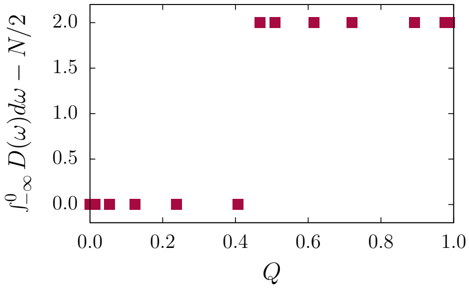

Supplementary Figure 6. Integrated density of states as a function of Q for open boundary conditions and $L = 36$.

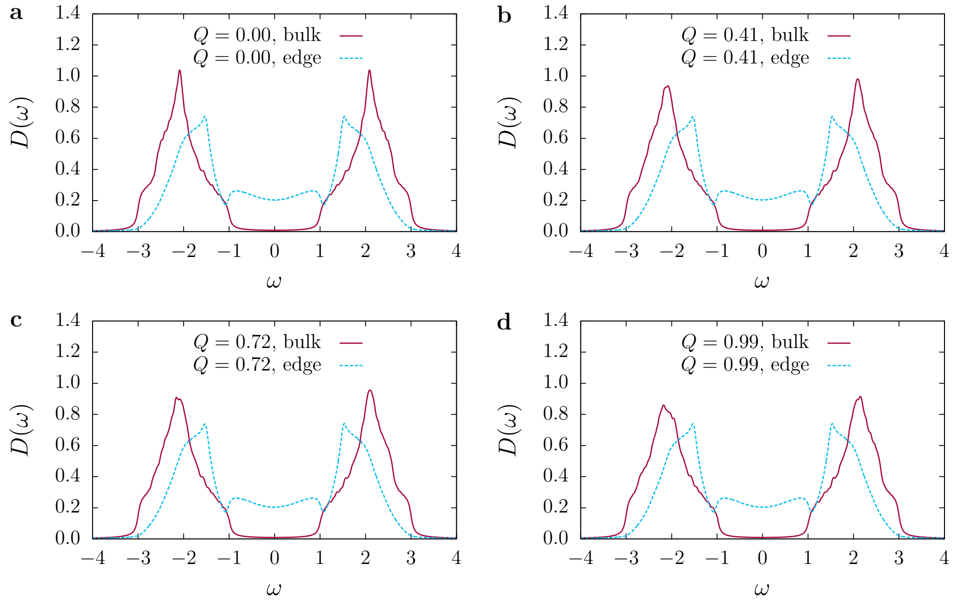

Supplementary Figure 7. Edge and bulk density of states for different $\boldsymbol{N}(\boldsymbol{x})$, corresponding to **a** $Q = 0$, **b** $Q = 0.41$, **c** $Q = 0.72$ and **d** $Q = 0.99$. Here, $L = 36$, and $D(\omega)$ was broadened as explained in Supplementary Fig. 5.

VI. SUPPLEMENTARY REFERENCES

[1] Parisen Toldin, F., Hohenadler, M., Assaad, F. F. & Herbut, I. F. Fermionic quantum criticality in honeycomb and π-flux Hubbard models: Finite-size scaling of renormalization-group-invariant observables from quantum Monte Carlo. *Phys. Rev. B* **91**, 165108 (2015).

[2] Senthil, T., Vishwanath, A., Balents, L., Sachdev, S. & Fisher, M. P. A. Deconfined Quantum Critical Points. *Science* **303**, 1490–1494 (2004).

[3] Gross, D. J. & Neveu, A. Dynamical symmetry breaking in asymptotically free field theories. *Phys. Rev. D* **10**, 3235–3253 (1974).

[4] Grover, T. & Senthil, T. Topological Spin Hall States, Charged Skyrmions, and Superconductivity in Two Dimensions. *Phys. Rev. Lett.* **100**, 156804 (2008).
